# Genome sequence of *Ensifer adhaerens* OV14 provides insights into its ability as a novel vector for the genetic transformation of plant genomes

**DOI:** 10.1186/1471-2164-15-268

**Published:** 2014-04-07

**Authors:** Steven Rudder, Fiona Doohan, Christopher J Creevey, Toni Wendt, Ewen Mullins

**Affiliations:** 1 Department of Crop Science, Teagasc Crops Research Centre, Oak Park, Carlow, Ireland; 2 UCD Earth Institute and UCD School of Biology and Environmental Sciences, University College Dublin, Belfield, Dublin 4, Ireland; 3 Animal and Bioscience Research Department, Animal and Grassland Research and Innovation Centre, Teagasc, Grange, Dunsany, Co. Meath, Ireland; 4 Current address: Institute of Biological, Environmental and Rural Sciences, Aberystwyth University, Aberystwyth, Ceredigion SY23 3FL, UK; 5 Current address: Carlsberg Research Centre, Gamle Carlsberg Vej 4-10, 1799 Copenhagen V, Denmark

**Keywords:** *Ensifer adhaerens*, Transformation, *Agrobacterium tumefaciens*, Genome sequencing

## Abstract

**Background:**

Recently it has been shown that *Ensifer adhaerens* can be used as a plant transformation technology, transferring genes into several plant genomes when equipped with a Ti plasmid. For this study, we have sequenced the genome of *Ensifer adhaerens* OV14 (OV14) and compared it with those of *Agrobacterium tumefaciens* C58 (C58) and *Sinorhizobium meliloti* 1021 (1021); the latter of which has also demonstrated a capacity to genetically transform crop genomes, albeit at significantly reduced frequencies.

**Results:**

The 7.7 Mb OV14 genome comprises two chromosomes and two plasmids. All protein coding regions in the OV14 genome were functionally grouped based on an eggNOG database. No genes homologous to the *A. tumefaciens* Ti plasmid *vir* genes appeared to be present in the OV14 genome. Unexpectedly, OV14 and 1021 were found to possess homologs to chromosomal based genes cited as essential to *A. tumefaciens* T-DNA transfer. Of significance, genes that are non-essential but exert a positive influence on virulence and the ability to genetically transform host genomes were identified in OV14 but were absent from the 1021 genome.

**Conclusions:**

This study reveals the presence of homologs to chromosomally based *Agrobacterium* genes that support T-DNA transfer within the genome of OV14 and other alphaproteobacteria. The sequencing and analysis of the OV14 genome increases our understanding of T-DNA transfer by non-*Agrobacterium* species and creates a platform for the continued improvement of *Ensifer*-mediated transformation (EMT).

## Background

The ability of *Agrobacterium tumefaciens* to transfer DNA into a plant cell via horizontal gene transfer has been instrumental in progressing the field of plant molecular biology, enabling methods such as T-DNA tagging [1,2], *Agrobacterium*-mediated transformation (AMT) for delivery of gene expression and silencing vectors [3,4], and the introduction of genes of interest into plant genomes [5]. In effect, these abilities have underpinned the integration of crop biotechnology into mainstream agriculture, driving the development of genetically modified crop varieties equipped with novel traits, which in 2013 were planted across 175 million hectares [6]. Indeed, based on the use of AMT, commodity crop improvement through genetic engineering has become the fastest adopted crop technology in the world with global value of the biotech/GM seed market estimated to be in excess of $13 billion [6]. However, the complexity of the *Agrobacterium* patent landscape remains a challenge for non-patent holders [7,8], as the execution of existing patents on crop biotechnology can restrict the widespread application of AMT technology by non-patent holders [9].

The possibility of modifying non-*Agrobacterium* strains to facilitate horizontal gene transfer was first described by Hooykaas et al. (1977), with work by van Veen et al. [10] showing that while *Phyllobacterium myrsinacearum* (harbouring the *A. tumefaciens* tumour inducing (Ti) plasmid) could cause tumorigenesis on plants, *Rhizobium meliloti* could not. It was not until 2005 though that the potential of non-*Agrobacterium* species’ to horizontally transfer genes into plant genomes was re-visited through CAMBIA’s Transbacter™ Project. Using the rhizobial species *Sinorhizobium meliloti* 1021, *Rhizobium* sp. NGR234 (now *Sinorhizobium fredii* NGR234) and *Mesorhizobium loti* MAFF303099, it was demonstrated that non-*Agrobacterium* rhizobia could indeed transfer T-DNA into plant cells [7]. However, the transformation frequency of these species was inadequate to provide a viable alternative to *A. tumefaciens*[11], which prompted the search for alternatives from a collection of diverse soil bacteria [11,12]. This initiative unearthed a lesser known rhizobial species, *Ensifer adhaerens*[13], as a rhizosphere inhabiting bacterium with the ability to successfully transform potato, tobacco and *Arabidopsis*. Designated *Ensifer adhaerens* OV14 (OV14), this strain can deliver sufficient transformation frequencies to present *Ensifer*-mediated transformation (EMT) as a viable alternative to existing transformation technology platforms [12].

The genetic and molecular mechanisms supporting the stable integration of *A. tumefaciens* T-DNA (*t*ransfer-DNA) into plant genomes have been the focus of intense research efforts since the first reports of AMT in the 1980s. A bacterial pathogen that causes ‘crown gall’ disease across a broad range of dicotyledonous and (some) monocotyledonous species [14], *A. tumefaciens* genetically transforms its host by transferring a single stranded DNA fragment (T-DNA) from its Ti plasmid into the host cell genome [15,16]. The T-DNA is exported from the bacterial cell into the plant cell together with several virulence effector proteins via a Type IV secretion system. By coating the T-DNA on its journey into the plant cell nucleus, this T-DNA structure appears more as a protein complex than a single strand of DNA [17]. For the purposes of genetic transformation, existing bacterial sequences within the left and right border of the T-DNA can be replaced with genes of interest (e.g. sequences coding for herbicide tolerance/disease resistance/synthesis of therapeutics), which can then be delivered into the targeted host genome using AMT. The reader is directed to a number of excellent reviews for an in-depth explanation and discussion of this process [18-20].

The genome sequences of *A. tumefaciens C58* (C58) and *S. meliloti 1021* (1021) were completed in 2001 [21-23]. Although these two gram-negative alphaproteobacteria are members of the same phylogenetic family (the Rhizobiaceae) and inhabit the rhizosphere, they operate very different lifestyles (pathogen vs. symbiont, respectively). The primary circular chromosomes of C58 and 1021 have been shown to share large-scale synteny, while only limited stretches of synteny can be found among additional replicons [24]. It is upon these more unique replicons that genes encoding functions leading to the different lifestyles of these organisms are found. For example, the above-mentioned T-DNA transfer mechanism of *A. tumefaciens* is located on the large Ti plasmid and genes key to the symbiotic interaction of 1021 with legumes are found on two megaplasmids namely pSymA and pSymB [25,26].

The application of functional genomic studies to dissect the processes of AMT have identified a number of genes located on the *A. tumefaciens* circular and linear chromosomes that are implicated in virulence through the processes of attachment, *vir* gene regulation, and resisting plant defence responses. Initial reversible attachment to plant cells involving beta-1,2-glucan and secondary irreversible attachment involving cellulose fibrils are early requirements *for A. tumefaciens* virulence while beta-1,2-glucan in *S. meliloti* plays an important role in symbiosis [27-30]. While the pAtC58 plasmid is non-essential for virulence of *A. tumefaciens*, it contains several *att* genes involved in attachment and pAtC58’s presence has been shown to have a positive effect on *vir* gene expression [31]. Mutations to a group of *chv* genes plus *ros*, *aopB* and *miaA* have all been shown to restrict, and in some cases halt virulence [32-37]. The ability of the bacterial cell to protect itself against plant derived reactive oxygen species (ROS) is also required for virulence by both plant pathogens and symbionts [38,39]. For example, a catalase (KatA) conferring gene has been shown to be upregulated in response to H_2_O_2_ via the peroxide sensor OxyR in both C58 and 1021 [38,40]. The rhizosphere is typically an acidic environment (~pH5.5) enriched by plant exudates including but not limited to sugars, ions, free oxygen and water [41,42]. Transcriptomic profiling of C58 and 1021 in response to a shift to acidic pH, has revealed a shared regulational change in genes involved in membrane composition and motility [42,43]. Separately, a chromosomally located two component sensor gene key to virulence in C58 and to symbiosis in 1021 termed chvG(exoS)/chvI has been cited as a global pH regulator [44]. A recently published study of 48 *Sinorhizobium* strains concluded that subtle differences in the presence of symbiosis associated genes involved in Nod-factor and polysaccharide biosynthesis, denitrification and Type III, IV, and VI secretion systems leads to varying compatibility among strains in legume-*Sinorhizobium* interactions [45]. An independent study looking at 14 rhizobia strains, including 1021, noted differences in gene content in key groups of genes, including those involved in nodulation, nitrogen fixation, production of exopolysaccharides and Type I to Type VI secretion systems with the authors concluding no simple ‘core symbiome’ exists among rhziobia [46]. In contrast to the number of comparative studies focused on symbiotic interactions that have been carried out to date, no study has yet focused on the ability of plant transformation within the rhizobia.

While a draft genome of *E. adhaerens* CSBa has recently been reported, to the best of our knowledge only DNA sequences for cobalamin biosynthetic (*cob*) genes are publicly available [47]. In this study the genome of OV14 was sequenced and functionally annotated by comparing to the already sequenced genomes of C58 and 1021 using the eggNOG database [48,49]. Subsequently, the literature was screened for all genes reported to have a positive effect on *A. tumefaciens* virulence and then homologs to these genes were sought for in OV14, and also in 1021 for additional comparison. The level of homology between genes was compared and, where relevant, gene copy number was considered. In addition, a phylogenetic analysis was completed on a core group of housekeeping genes and *Rhizobiales* chromosomal-located virulence related genes to clarify the position of OV14 within the large, diverse Rhizobiaceae family.

## Results

### General features of the E. adhaerens OV14 genome versus that of A. tumefaciens C58 and S. meliloti 1021

**Figure 1:**
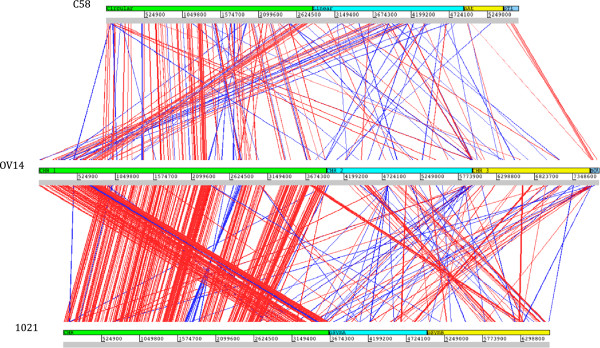
**Comparative synteny plots showing total genome content of *Agrobacterium tumefaciens *C58 (top bar), *Ensifer adhaerens *OV14 (middle bar), and *Sinorhizobium meliloti *1021 (bottom bar), computed using DoubleACT version2 on tBLASTx setting.** Visualised in Artemis ACT with cut off set at 1000. The replicons within each genome are separated by coloured bars and labelled. Homology between the genomes is displayed via interconnecting lines; red lines representing direct homology with blue lines corresponding to inverted homologous sequence.

The OV14 genome is the largest of the three species at 7.71 Mb; 2.04 Mb bigger than the C58 genome and 1.01 Mb larger than the 1021 genome (Table 1). Composed of four replicons of sizes 3.96 Mb, 2.01 Mb, 1.61 Mb and 125 kb, the OV14 genome is similar to that of 1021, which is also made up of three large circular replicons minus the small accessory plasmid. In contrast the C58 genome differs dramatically with the circular chromosome being approximately 25% less than the size of OV14’s and 1021’s counterpart and the presence of a linear chromosome being a feature unique to C58. That said, OV14 (Additional file 1: Figure S1) and C58 share a similar sized 120–180 kb mobile plasmid not found in 1021. The GC content of C58’s genome is notably lower (at 58%) compared to that of OV14 (60.75%) and 1021 (62.17%) genomes (Table 1), with a total genome comparison highlighting a greater level of synteny between OV14 and 1021 (Figure 1).

**Table 1:** **Basic genome information for three species; *Ensifer adhaerens *** **OV14, *Sinorhizobium meliloti *** **1021, and *Agrobacterium tumefaciens *** **C58**

**OV14**	**1021**	**C58**
CHR 1	CHR	CHR Circular
3956045 bp	3654135 bp	2841580 bp
916 average gene length	939 average gene length	902 average gene length
62.24 GC %	62.7 GC %	59.4 GC %
9 rRNA	3 rRNA	2 rRNA
52 tRNA	51 tRNA	40 tRNA
CHR 2	pSymA	CHR Linear
2012811 bp	1354226 bp	2075577 bp
916 average gene length	875 average gene length	988 average gene length
61.77 GC %	60.4 GC %	59.3 GC %
3 rRNA	0 rRNA	2 rRNA
4 tRNA	2 tRNA	13 tRNA
pOV14b	pSymB	pAt
1614950 bp	1683333 bp	542868 bp
860 average gene length	949 average gene length	849 average gene length
60.65 GC %	62.4 GC %	57.3 GC %
3 rRNA	0 rRNA	0 rRNA
4 tRNA	1 tRNA	0 tRNA
pOV14c		pTi
125203 bp		214233 bp
815 average gene length		938 average gene length
58.37 GC %		56.7 GC %
0 rRNA		0 rRNA
0 tRNA		0 tRNA

For each entry information is as follows; Replicon id, Replicon size, average gene length, Replicons GC content as percentage, No. rRNA, and No. tRNA.

Structurally, chromosome 1 of OV14 shares 54% nucleotide homology with the chromosome of 1021, while the circular and linear chromosomes of C58 share 20% and 5% homology, respectively with chromosome 1 of OV14. Chromosome 2 of OV14 shows reduced homology of 20% to pSymB, 2% to pSymA, and 3% to the 1021 chromosome. In regards to C58, 3% and 7% homology to chromosome 2 of OV14 is noted for the circular and linear chromosome, respectively. The third replicon of OV14 shows 7% homology to both the pSymA and pSymB of 1021, and 4% homology to the linear chromosome of C58. Finally the small accessory plasmid pOV14c shows between 1–2% homology to each of the replicons. Homology of pOV14 to C58 replicons are found in pTi and pAt at 22% and 3%, respectively.

**Figure 2:**
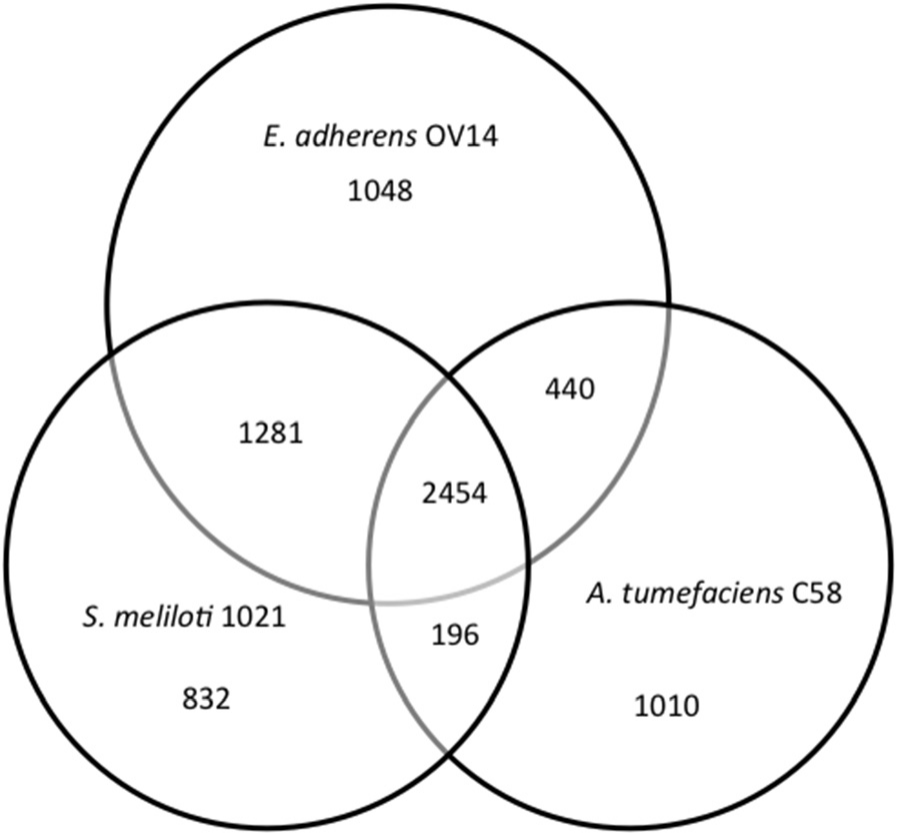
**Venn diagram illustrating the number of eggNOGs found across the three species. ***Ensifer adhaerens* OV14, *Sinorhizobium meliloti* 1021, and *Agrobacterium tumefaciens* C58.

The three genomes of OV14, 1021 and C58 were compared by using evolutionary genealogy of genes: Non-supervised Orthologous Groups (eggNOG) assignments. The eggNOG database is formatted to functionally categorise genes within twenty-five groups. Twenty-one of the 25 eggNOG functional categories have representatives in the three genomes in this study (Table 2). Those categories that are not represented are RNA processing and modification [A], nuclear structure [Y], cytoskeleton [Z] and extracellular structures [W]. In total 7261 NOGs were identified in this study with 2454 (33.8%) being shared among the three species (Table 2). OV14 has the most species-specific NOGs at 1048 (14.4%), marginally ahead of C58 with 1010 (13.9%) whilst 1021 has less at 832 (11.5%). Of the NOGs that are shared between two species and not present within the third species; OV14 and 1021 share 1281 (17.6%) almost 3-fold more than that shared by OV14 and C58 at 440 (6.1%), and 6.5-fold more than that shared between C58 and 1021 at 196 (2.7%) (Figure 2).

**Table 2:** **Comparison of eggNOG assignments for *Ensifer adhaerens *** **OV14, *Agrobacterium tumefaciens *** **C58, and *Sinorhizobium meliloti *** **1021**

**eggNOG functional category**	**Shared by 3 species**	**OV14 & C58 only**	**OV14 & 1021 only**	**C58 & 1021 only**	**OV14 only**	**C58 only**	**1021 only**
Information storage and processing							
[J] Translation, ribosomal structure and biogenesis	143	4	18	3	10	14	10
[K] Transcription	160	58	135	19	140	116	83
[L] Replication, recombination and repair	99	16	40	7	42	27	37
[B] Chromatin structure and dynamics	1	0	0	0	1	0	0
Cellular processes and signaling							
[D] Cell cycle control, cell division, chromosome partitioning	22	2	5	0	2	6	5
[V] Defense mechanisms	28	4	12	3	17	11	12
[T] Signal transduction mechanisms	83	30	49	5	35	45	35
[M] Cell wall/membrane/envelope biogenesis	119	16	59	5	34	49	30
[N] Cell motility	31	2	5	0	2	4	3
[U] Intracellular trafficking, secretion, and vesicular transport	34	13	13	10	16	13	9
[O] Posttranslational modification, protein turnover, chaperones	112	8	29	2	21	13	17
Metabolism							
[C] Energy production and conversion	164	21	82	14	52	55	58
[G] Carbohydrate transport and metabolism	194	38	121	17	76	61	59
[E] Amino acid transport and metabolism	317	51	128	27	120	118	61
[F] Nucleotide transport and metabolism	67	7	14	3	7	7	5
[H] Coenzyme transport and metabolism	97	6	29	3	10	12	9
[I] Lipid transport and metabolism	96	9	48	9	32	36	26
[P] Inorganic ion transport and metabolism	140	23	46	11	35	57	41
[Q] Secondary metabolites biosynthesis, transport and catabolism	56	12	45	6	36	15	31
Poorly characterized							
[R] General function prediction only	151	39	90	14	97	80	56
[S] Function unknown	340	81	313	38	263	271	245

Five of the functional categories grouped under the heading of cellular processing and signalling were of most interest to this study (Table 2). In category [V], ‘defence mechanisms’, a total of 87 NOGs were recorded with 28 (32.2%) shared across all three species. Within this category OV14 recorded the most individual NOGs at 17 (19.5%) followed by 1021 and C58 with 12 (13.8%) and 11 (12.6%), respectively. For the signal transduction mechanisms [T] category a total of 282 NOGs were found of which 83 (29.4%) were shared by all species, while within this category C58 contained 45 individual NOGs, 10 more than both OV14 and 1021. Also to be noted were the minimal number of NOGs shared by C58 and 1021 (n = 5) compared to OV14 and C58 (n = 30) and OV14 and 1021 (n = 49). Within the category cell wall/membrane/envelope biogenesis [M] 119 (38.2%) of a total of 312 were shared by all three species; OV14 and 1021 sharing 59 (19%), which was more than the 16 (5%) shared across OV14 and C58 and the 5 (1.6%) for C58 and 1021. Although C58 shares a lower number of NOGs with the other two species in category [M] it does possess the highest number of species specific NOGs at 49 (15.7%). Cell motility is category [N] representing 47 NOGs for which 31 (66%) were shared by all three species, with no NOGs shared by C58 and 1021. In category [U] (Intracellular trafficking, secretion, and vesicular transport) there were a total of 108 NOGs of which 34 (31.5%) were shared by all three species. Category [U] showed the most even distribution of any category. The final category of specific interest is post-translational modification, protein turnover, chaperones [O] with a total of 202 NOGs for which 112 (55.4%) were shared by all three species; OV14 and 1021 with 29 (14.4%), OV14 and C58 and C58 and 1021 with 8 (4%) and 2 (1%), respectively. Individually, OV14 possessed 21 (10.4%), 1021 possessed 17 (8.4%) and C58 possessed 13 (6.4%).

### Attachment

The chromosomal virulence gene A (*chvA*) is a member of a group of orthologous genes found in the eggNOG database under the code aproNOG01094 (Table 3). Encoding a cyclic beta-1,2-glucan ABC transporter, its function is linked to *chvB* a member of aproNOG01088, which encodes a cyclic beta 1-2 glucan synthase. Together *chvA* and *chvB* function to synthesise and transport beta-1,2 glucan across the inner membrane, which is required for attachment of the bacterial cell to the plant cell surface. Genes with parallel function named *ndvA* and *ndvB* are found in 1021. All three species were found to possess one gene in the aproNOG01094 representative of *chvA*/*ndvA* (Table 3). Located on chromosome one of OV14 is a gene showing 89% protein sequence identity to *ndvA* of 1021 and 77% identity to *chvA* of C58. Two genes downstream of OV14’s *chvA*/*ndvA* homolog was a *chvB*/*ndvB* homolog (a member of aproNOG01088) showing 86% and 68% protein sequence identity to respective sequences in 1021 and C58, respectively. OV14 also has a second *chvB*/*ndvB* gene sharing 47% and 50% protein sequence identity to 1021’s and C58’s *chvB*/*ndvB* respectively. A third gene involved in the synthesis of beta 1-2 glucan is *pscA*/*exoC* (aproNOG01465) encoding a phosphoglucomutase, which recorded 90% and 81% to the respective target in 1021 and C58, respectively.

**Table 3:** **Comparative analysis for the presence/absence of genes identified to have a positive effect on *Agrobacterium *** **virulence that are located in the genomic background (not on Ti plasmid) of *Ensifer adhaerens *** **OV14, *Agrobacterium tumefaciens *** **C58, and *Sinorhizobium meliloti *** **1021**

**Gene id**	**eggNOG id**	**OV14 gene**	**C58 gene**	**1021 gene**	**No. species**	**No. proteins**	**Virulence function**	**Product**
**In C58**		**Copy number**	**Copy number**	**Copy number**	**In NOG**	**In NOG**	**In C58**	
*chvA*	aproNOG01094	1	1	1	52	52	Attachment	Cyclic beta-1,2-glucan ABC transporter
*chvB*	aproNOG01088	2	1	1	39	40	Attachment	Cyclic beta 1-2 glucan synthase
*pscA(exoC)*	aproNOG01465	1	1	1	65	65	Attachment	Phosphoglucomutase
*pcs*	aproNOG02893	1	1	1	37	37	Attachment	Phosphatidylcholine synthase
*pmtA*	aproNOG06650	1	1	1	58	58	Attachment	Phospholipid N-methyltransferase
*choX*	aproNOG00993	2	2	2	35	41	Attachment	Choline SBP
*choW*	aproNOG00971	1	1	1	35	35	Attachment	Choline ABC ATPase
*choV*	aproNOG02245	2	3	1	45	78	Attachment	Choline ABC permease
*chvD*	aproNOG00260	1	1	1	102	104	*vir* gene regulation	Uracil phosphoribosyltransferase
*chvE*	aproNOG03985	1	1	1	29	20	*vir* gene regulation	Multiple sugar-binding periplasmic receptor
*chvG*	aproNOG00593	1	1	1	86	89	*vir* gene regulation	DNA-binding/iron metalloprotein/AP endonuclease
*chvI*	aproNOG03091	1	1	1	85	87	*vir* gene regulation	Transcriptional regulator
*chvH*	aproNOG03687	1	1	1	50	50	*vir* gene regulation	Elongation factor P
*miaA*	aproNOG00010	1	1	1	116	116	*vir* gene regulation	tRNA delta (2)-isopentenylpyrophosphate transferase
*ros*	aproNOG09171	1	1	1	16	17	*vir* gene regulation	Ros/MucR family transcriptional regulator
*picA*	aproNOG09265	1	2	0	13	15	Host cell wall degradation	Polygalacturonase
*kdgF*	aproNOG11632	1	1	1	15	15	Host cell wall degradation	Pectin degradation protein
*ligE*	aproNOG03997	1	1	1	31	31	Host cell wall degradation	Lignin degradation protein
*acvB*	aproNOG05730	1	1	2	28	31	Forms complex with T-strand	Acid tolerance and virulence protein
*aopB*	aproNOG08879	1	1	1	17	17	Defence	Porin-like membrane protien
*katA*	aproNOG00015	2	1	1	51	54	Defence	Catalase-Peroxidase
*dps*	aproNOG08385	0	1	0	19	19	Defence	DNA starvation/stationary phase protection protein
*catE*	aproNOG02507	0	1	1	40	40	Defence	Catalase
*oxyR*	aproNOG01190	1	1	0	34	48	Defence	Oxidative stress transcription regulator
*oxyR* (1021)	aproNOG01330	0	0	1	33	33	Defence	Oxidative stress transcription regulator
*sodB*	aproNOG00877	2	3	1	115	122	Defence	Super oxide dismutase

The OV14 genome was also screened for the presence/absence of genes linked to the C58 pAt *att* locus, which contains up to 24 genes for which some have been implicated in the early stages of attachment and virulence. The *attR* gene is part of aproNOG01724 and encodes an acetyltransferase but no copies were found across the OV14 genome, compared to a single copy in 1021 and two *attR* copies in C58. The genes *attB* and *attD* are implicated in bacteria-plant signalling during root colonisation and at the wound site during pathogenesis, with *attB* part of aproNOG06835 annotated as part of a binding-protein-dependent transport system, which is predicted to transport mannopine in C58 [50]. While 1021 and C58 both possess a single copy, a member of the aproNOG06835 was not found in OV14. The *attD* gene of C58 appears to be unique, unassigned to any aproNOG and having no similar sequence in OV14 or 1021. Mutations to genes *attC* and *attG* can render *Agrobacterium* avirulent on tomato and carrot by preventing attachment to the host cell [51]. Both genes are annotated as ABC transporters for which no orthologs exist in OV14: an *attC* ortholog in aproNOG06683 is present in 1021 and C58 (Table 4). The *attKLM* (renamed *blcABC*) operon within this locus has been linked to quorum sensing and found to be up regulated in response to salicylic acid (SA) in C58 [52]. All three species contain multiple gene entries (four copies in OV14, three in C58 and five in 1021) in aproNOG00713, which houses the C58 *blcA* (a NAD-dependent succinyl dehydrogenase). No genes homologous to C58 *blcB* (aproNOG04363) or *blcC* (aproNOG02812) were found in OV14. While the role of the remaining genes of the *att* locus in virulence remains unclear, the genes *attE*, *attF*, *attG*, *attH*, *attO*, *attT*, *attV*, *attY* and *attZ* were not found within the OV14 genome, or that of 1021 either (Table 4). However, they are represented by aproNOGs highlighting their presence among other alphaproteobacteria. Genes found in the C58 *att* locus not represented by aproNOGs include *attD*, *attP*, *attS*, *attU*, *attW*, and *attX*.

**Table 4:** **Comparative analysis for the presence/absence of genes involved in attachment to plant cell that are located in the genomic background (not on Ti plasmid) of *Ensifer adhaerens *** **OV14, *Agrobacterium tumefaciens *** **C58, and *Sinorhizobium meliloti *** **1021**

**Gene id**	**eggNOG**	**OV14 gene**	**C58 gene**	**1021 gene**	**No. species**	**No. protein**	**Virulence function**	**Product**
**In C58**		**Copy number**	**Copy number**	**Copy number**	**In NOG**	**In NOG**	**In C58**	
*celD*	aproNOG11524	0	1	0	7	7	Attachment	Cellulose biosynthesis protein
*celE*	aproNOG04598	1	1	0	7	7	Attachment	Cellulose synthesis protein
*celG*	aproNOG12411	1	1	0	23	26	Attachment	Cellulose synthesis protein
*celC*	aproNOG07630	1	1	0	25	28	Attachment	Endoglucanase
*celB*	aproNOG09454	1	1	0	23	26	Attachment	Cellulose synthase
*celA*	aproNOG05761	1	1	0	27	30	Attachment	Cellulose synthase
*attK2*	aproNOG02908	0	1	1	17	18	Attachment	Semialdehyde dehydrogenase
*attA1*	aproNOG00407	3	2	4	47	73	Attachment	ABC transporter, nucleotide binding/ATPase protein [putrescine]
*attA2*	aproNOG05011	0	1	1	15	15	Attachment	ABC transporter, membrane spanning protein [mannopine]
*attB*	aproNOG06835	0	1	1	16	16	Attachment	ABC transporter, membrane spanning protein [mannopine]
*attC*	aproNOG06683	0	1	1	12	12	Attachment	ABC transporter, substrate binding protein [mannopine]
*attE*	aproNOG05033	0	2	0	24	25	Attachment	ABC transporter nucleotide binding/ATPase protein
*attF*	aproNOG00433	0	3	0	24	26	Attachment	ABC transporter, membrane spanning protein
*attG*	aproNOG00433	0	3	0	24	26	Attachment	ABC transporter, membrane spanning protein
*attH*	aproNOG04763	0	2	0	22	23	Attachment	Hypothetical protein
*attJ/blcR*	aproNOG06067	0	1	0	17	17	Attachment	Transcriptional repressor of the blcABC operon
*attK/blcA*	aproNOG00713	4	3	5	88	141	Attachment	NAD-dependent succinyl-semialdehyde dehydrogenase
*attL/blcB*	aproNOG04363	0	1	1	15	19	Attachment	Gamma hydroxybutyrate dehydrogenase
*attM/blcC*	aproNOG02812	0	2	0	19	23	Attachment	Zn-dependent gamma butyryl lactone lactonase
*attO*	aproNOG09383	0	1	0	5	5	Attachment	Transcriptional regulator, AraC family
*attR*	aproNOG01724	0	2	1	24	27	Attachment	Transacetylase
*attT*	aproNOG12782	0	1	0	3	3	Attachment	GNAT family acetyltransferase
*attV*	aproNOG09482	0	1	0	12	12	Attachment	Mg (2+) transport ATPase
*attY*	aproNOG07710	0	1	0	45	48	Attachment	Glutathione S-transferase
*attZ*	aproNOG08980	0	1	0	21	26	Attachment	Transcriptional regulator

The *cel* locus is comprised of six genes *celABCDEG* and encodes a synthase for cellulose fibrils implicated in the second stage of attachment referred to as tight binding, which is irreversible [53] and critical for the virulence of *Agrobacterium* cells [53]. The genome of OV14 had genes orthologous to *celABCEG*, but not *celD*, which are thought to be cytoplasmic lipid carriers (Table 4). The aproNOGs representing *celABCG* genes were found in 23–27 separate alphaproteobacteria while aproNOGs including *celDE* were located in only 7 alphaproteobacteria. Of interest, 1021 did not contain any orthologs to the *cel* genes of C58 (Table 4).

The presence of phosphatidylcholine in prokaryotic membranes is generally confined to species that intimately interact with eukaryotic cells [54]. Two pathways present in C58 can lead to phosphatidylcholine production; the methylation pathway that requires *pmtA* (aproNOG06650) and the pcs pathway that requires *pcs* (aproNOG02893) [54]. In C58 phosphatidylcholine is found in the inner and outer membrane constituting around 23% of total membrane lipids. The *pcs* gene of OV14 shares 92% and 85% protein sequence identity with 1021 and C58 respectively, while the *pmtA* gene of OV14 shares 83% and 67% protein sequence identity with 1021’s and C58’s respecitvely. The pcs pathway is dependent on the uptake of choline from the environment [55]. Screening OV14 for the choline ABC transporter genes *choXWV* (that have been identified in both C58 and 1021), revealed that the *choX* solute binding protein component (aproNOG00993) was represented by two orthologs in all three species (Table 3). The ABC ATPase *choW* (aproNOG00971) has one member in each species and the choline permease (aproNOG02245) was noted to have three members in C58, two in OV14 and one in 1021.

### Host cell wall degradation

The C58 genome contains two copies of the *picA* gene (aproNOG09265), which encodes a polygalacturonase to degrade the pectin network in targeted cell walls and aid the secretion of bacterial proteins into the plant cell [56]. The genome of OV14 was equipped with one copy showing 78% identity to *picA* of C58, while 1021 has no recorded *picA* homolog (Table 3). A complementary gene involved in pectin degradation is *kdgF* (aproNOG11632), with all three species possessing a *kdgF* homolog; OV14 sharing 72% and 47% protein sequence identity with C58 and 1021 respectively. Finally all three species possessed a member of the aproNOG03997, a beta-etherase linked to a lignin degradation protein annotated as *ligE* in 1021 and C58, with OV14’s *ligE* homolog sharing 77% and 65% protein sequence identity to 1021 and C58, respectively.

### Chromosomal regulation of Ti based virulence genes

Key to the regulation of *vir* genes in *Agrobacterium* is the chvG/chvI two-component sensor, with a mutation to either *chvG* or *chvI* halting virulence [34]. Responsive to acidic pH, the chvG/chvI sensor regulates *aopB* and *katA*, two genes involved indirectly in virulence by promoting homeostasis in acidic conditions. A homologous system in 1021 is also responsive to acidic pH. This two component sensor encoded by *exoS/chvI* regulates the production of succinoglycan and is vital for symbiosis with alfalfa [57]. The OV14 genome has genes homologous to both *chvG* (*exoS*) (aproNOG00593) and *chvI* (aproNOG03091) situated in an operon as expected for two-component sensors (Table 3). The OV14 *exoS* (*chvG*) homolog shared 91% and 79% protein sequence identity to 1021’s *exoS* and C58’s *chvG*, whereas the OV14 *chvI* homolog shared 94% and 91% protein sequence identity to 1021 and C58 *chvI* homologs, respectively. The chvD from C58 interacts with virB8 and has a positive effect on virulence, with both virulence and *vir* gene expression greatly reduced when the function of chvD is disrupted in C58 [32]. A member of aproNOG00260, all three species contained a homolog with the OV14 homolog recording 89% and 87% protein sequence identity, corresponding to that of 1021 and C58.

Chromosomal virulence gene E (*chvE*) codes for a multiple sugar binding periplasmic sensor, which interacts with the periplasmic domain of virA aiding in the regulation of the *vir* operon through the virA/G two-component sensor. OV14 possesses *chvE* as a member of the aproNOG03985, sharing 93% and 77% protein sequence identity to its 1021 and C58 counterparts. The *chvE* homologs of 1021 and OV14 are ~100 bp shorter than the version located in C58, with the difference found towards the N terminus of the gene where a putative ligand binding site is positioned. On the C58 circular chromosome, *chvE* is located adjacent to *gguABC* components of an ABC sugar transporter. The same operon arrangement is found in all three species *chvE*-*gguABC* with all species’ *gguABC* genes present in the same aproNOGs (A = aproNOG01497, B = aproNOG03238, and C = aproNOG05875). In regards to the C58 gene *chvH* (encoding elongation P factor, member of aproNOG03687), virulence of *A. tumefaciens* is decreased in the *chvH* mutant, which has been linked to reduced levels of virE2 [33]. The OV14 *chvH* homolog was found to share 95% and 84% protein sequence identity to 1021’s and C58’s respective copy.

The *A. tumefaciens ros* regulator (aproNOG09171) has been shown to repress the *virC* and *virD* operons by binding to a *ros* box within promoter regions of both genes, but the binding activity of virG is able to overcome this repression although the exact mechanism is unclear. The 1021 *ros* homolog named *mucR* (aproNOG09171) is involved in the regulation of both motility and exopolysaccharide production [58]. The OV14 genome has one gene homologous to the *ros* gene sharing 92% and 79% to 1021 (*mucR*) and C58 counterparts, correspondingly (Table 3). A homolog of the C58 *miaA* gene was found in OV14, with mutations of the *miaA* gene in *A. tumefaciens* reported to marginally decrease *virB*, *virD* and *virE* gene expression [36]. The *miaA* gene (aproNOG00010) encodes a tRNA delta (2)-isopentenylpyrophosphate transferase which is involved in protein translation; a homolog was also identified in 1021.

### Chromosomal based acvB

C58’s *acvB* (aproNOG05730) contains multiple annotations, the most common being an acid induced virulence protein and the virJ-like protein. The acvB protein has been reported to bind to the T-strand in the periplasm increasing transport efficiency to the plant cell compared to an *acvB*^*-*^strain [59]. In this regard, OV14 contained one entry in aproNOG05730 as did C58, while 1021 possessed two. The OV14 *acvB* homolog shares 53% protein identity to C58 *acvB* and the two 1021 *acvB* orthologs SMc00612 and SMc00613 were found to share disrupted homology to the C58 and OV14 *acvB* genes (Table 3).

### Protecting against plant defences

The C58 gene *katA* (aproNOG00015) encodes a catalase-peroxidase implicated in virulence through detoxification of hydrogen peroxide encountered during bacteria-plant interactions [39]. Three catalase genes have been previously identified in 1021; *katA*, *katB* and *katC*[38], with the 1021 *katB* being a member homologous to and a member of the same aproNOG as the C58 *katA*. OV14 had two gene members in aproNOG00015; one sharing 89% protein sequence identity with C58’s *katA* and 61% identity to *katB* of 1021 and a second more putative gene with 64% similarity to the C58 *katB* gene. The oxyR peroxide sensor regulates transcription of *katA* in C58 with hydrogen peroxide and superoxide anions indirectly/directly oxidizing oxyR leading to *katA* activation [40]. Although the *oxyR* gene of C58 and 1021 are found separately in aproNOG01190 and aproNOG01330 respectively, OV14 was found to only contain a homolog of the C58 *oxyR*. While OV14 does possess a dps family protein (aproNOG06937); not found in C58 or 1021. No homologs to the C58 *dps* (aproNOG08385), which functions to protect DNA from hydroxyl radicals produced during oxidation of Fe (II) by hydrogen peroxide [60] were detected in OV14, or 1021 (Table 3).

C58 and 1021 both have a single catalase gene in aproNOG02507 that functions in protecting cells from the toxic effects of hydrogen peroxide, annotated as *catE* in C58 and *catC* in 1021. No homolog was detected in OV14. Superoxide dismutases help to protect the cell via dismutation of superoxide into oxygen and hydrogen peroxide and three copies of the *sodB* gene (aproNOG00877) were found in C58, two copies in OV14 and one copy in 1021 (Table 3). Knockout of all three *sodB* genes in *A. tumefaciens* results in avirulence, while only the *sodBI* mutant shows reduced virulence when targeted individually [61].

### Ti based virulence

The *vir* operon found on the C58 Ti plasmid encodes the core machinery for the production and transport of T-DNA from the bacterial cell with the two-component regulator virA/G switching on expression of ancillary *vir* genes upon detection of plant phenolics. No homologs of this system were found to exist in OV14 (Additional file 1: Figure S2) or 1021. A combination of *virB* genes and *virD4* form the Type IV secretion system of C58. Part of aproNOG03383 (Table 5), the *virD4* aproNOG is shared by seventy-two species. The aproNOG03383 has two entries in C58 (Atu4858 and Atu6184), named *virD4*-like and *virD4*, respectively. Four *virD4*-like genes were identified in OV14. Upon inspection only one was found to share a protein sequence identity exceeding 50% with any known alphaproteobacteria gene, sharing 71% protein sequence identity to virD4 (Arad_15020) of *A. rhizogenes* K84.

**Table 5:** **Comparative analysis for the presence/absence of Ti-based virulence genes in *Ensifer adhaerens *** **OV14, *Agrobacterium tumefaciens *** **C58, and *Sinorhizobium meliloti *** **1021**

**Gene id**	**eggNOG**	**OV14 gene**	**C58 gene**	**1021 gene**	**No. species**	**No. protein**	**Virulence function**	**Product**
**In C58**		**Copy number**	**Copy number**	**Copy number**	**In NOG**	**In NOG**	**In C58**	
*virA*	aproNOG05576	0	1	0	7	7	*vir* operon regulation	Sensor kinase
*virG*	aproNOG04872	0	1	0	9	10	*vir* operon regulation	Regulatory protein
*virB1*	aproNOG10673	0	1	0	21	22	Type IV secretion	Type IV secretion system lytic transglycosylase
*virB2*	aproNOG12925	0	1	0	8	10	Type IV secretion	Type IV secretion system Pilin subunit protein
*virB3*	aproNOG08388	0	2	1	20	27	Type IV secretion	Type IV secretion system Pilin-like protein
*virB4*	aproNOG02013	2	2	1	71	117	Type IV secretion	Type IV secretion system ATPase
*virB5*	N/A	0	1	0	N/A	N/A	Type IV secretion	Type IV secretion system protein
*virB6*	N/A	0	1	0	N/A	N/A	Type IV secretion	Type IV secretion system protein
*virB7*	N/A	0	1	0	N/A	N/A	Type IV secretion	Type IV secretion system protein
*virB8*	N/A	0	1	0	N/A	N/A	Type IV secretion	Type IV secretion system protein
*virB9*	aproNOG01070	0	1	1	20	29	Type IV secretion	Type IV secretion system protein
*virB10*	aproNOG01880	0	2	1	25	32	Type IV secretion	Type IV secretion system ATP energy sensor
*virB11*	aproNOG02544	1	2	1	70	87	Type IV secretion	Type IV secretion system ATPase
*virC1*	aproNOG17216	0	1	0	6	6	Generation of the T-strand	DNA-binding protein
*virC2*	N/A	0	1	0	N/A	N/A	Generation of the T-strand	Hypothetical protein
*virD1*	aproNOG18795	0	1	0	5	5	T-DNA processing	Endonuclease accessory protein
*virD2*	aproNOG06745	0	1	0	13	16	T-DNA processing	Endonuclease
*virD3*	aproNOG10158	0	1	0	14	14	T-DNA processing	Hypothetical protein
*virD4*	aproNOG03383	1	2	0	72	121	Type IV secretion	Coupling protein
*virD5*	N/A	0	1	0	N/A	N/A	T-DNA processing	Hypothetical protein
*virE0*	N/A	0	1	0	N/A	N/A	Generation of the T-strand	Hypothetical protein
*virE1*	N/A	0	1	0	N/A	N/A	Generation of the T-strand	Chaperone protein
*virE2*	N/A	0	1	0	N/A	N/A	Generation of the T-strand	ss-DNA binding protein
*virE3*	N/A	0	1	0	N/A	N/A	Generation of the T-strand	Hypothetical protein
*virF*	N/A	0	1	0	N/A	N/A	Effector	Hypothetical protein
*virH1*	aproNOG14518	0	1	0	3	3	Non-essential	Hypothetical protein
*virH2*	aproNOG15187	0	1	0	3	3	Non-essential	Hypothetical protein
*virK*	aproNOG20065	0	1	0	4	4	Non-essential	Hypothetical protein

The *virB* operon encodes for eleven proteins (numbered 1–11), which form the T-DNA transporting type IV secretion system. C58 has three similar Type IV secretion systems, a Ti-plasmid based *virB*, a Ti plasmid *trb* operon and a linear chromosome based *avh. VirB3*, *virB4*, *virB10*, and *virB11* form part of the same aproNOGs as their *avh* counterparts, with *virB1*, *virB2*, and *virB9* found in different aproNOGs from counterpart’s *avhB1*, *avhB2*, *avhB11*. While *avhB5*, *avhB6*, avhB7, and *avhB8* are all found in aproNOGs, *virB5*, *virB6*, *virB7*, and *virB8* are not associated with any aproNOG. In this analysis, OV14 shares aproNOG02013 (housing *avhB3*), aproNOG04596 (housing *avhB6*) and aproNOG02544 (housing *avhB11*) only. The genes present from OV14 identified as part of aproNOG02013 showed less than 50% protein sequence identity to the closest matches from *Phenylobacterium zucineum* HLK1 and *Erythrobacter litoralis* HTCC2594, with OV14 containing 4 genes for aproNOG04596.

Some proteins encoded by the *vir* regulon are non-essential for transformation but are known to increase transformation efficiency. The respective proteins of *virC1* and *virC2* may enhance nicking at the right border of T-DNA and virE2 is exported to the plant cell along with the T-strand potentially protecting the ss-DNA from degradation or detection. The virE1 protein binds to virE2 within the bacterial cell blocking interaction with the T-strand until within the plant cell. No homologs for either were found in OV14 or 1021. The genes of the *virE* operon and *virC2* were not found in any aproNOG but the *virC1* gene was a member of aproNOG17216 that was identified to be present in 6 alphaproteobacteria species. The virulence genes *virF*, *virH1* and *virH2* have been implicated in the expansion of the host range during *Agrobacterium*-mediated transformation [22], with virF involved in the stripping of virE2 proteins off the T-DNA and virH1 and virH2 involved in the detoxification of anti-bacterial phenolics. With gene *virF* not part of an aproNOG and *virH1* and *virH2* of C58 sharing aproNOGs with only 2 other species, *Agrobacterium rhizogenes* K84 and *Chelativorans* sp. BNC1, no homologs were found in OV14 or 1021.

### Type IV secretion systems

Compared to the three T4SS found in C58 (based on *virB*, *avhB*, and *trb*), only one T4SS was identified in OV14 (based on *trb*); equivalent to a single system also present in 1021 (based on *avhB*). The virB T4SS, which is known to export T-DNA from the bacterial cell into the plant cell is only found on the Ti-plasmid of *A. tumefaciens*. C58’s trb is known to be responsible for conjugation of the Ti-plasmid between bacterial cells. A homologous trb was found on pOV14c (Additional file 1: Figure S2), with the *trb* operon sharing the same gene arrangement and located immediately upstream of *repABC* in both the OV14 and C58 genome. The protein sequence identities of the 11 genes comprising the *trb* operon ranged from 71% to 90%.

### pH responsive gene networks

Key to *vir* gene regulation is *chvI*, which functions with *chvG* (known as *exoS* in *S. meliloti* 1021) and is up regulated in both C58 and 1021 [42,43]. Nine *exo* genes (*exoF*, *exoH*, *exoK*, *exoL*, *exoN*, *exoQ*, *exoT*, *exoW*, and *exoY*) involved in the synthesis and metabolism of succinoglycan are shared and up regulated in both organisms and all nine genes were found to be present in OV14. Two additional *exo* genes (*exoM* and *exoU*) were also noted to be in all three species. The acid inducible membrane protein aopB (aproNOG08879), which is involved in C58 virulence was also present as a single copy in OV14 and 1021 (ropB1) (Table 3). All three species are represented in this NOG with the OV14 homolog sharing 55% and 82% protein sequence homology with the C58 *aopB* and 1021 *ropB1*, respectively. Finally a recently identified *imp* type VI (T6SS) secretion system which is up regulated in C58 in response to low pH (5.5) [42] was not found in OV14 nor 1021. The function of this T6SS in C58 has yet to be determined.

### Phylogenetic positioning of *E. adhaerens* OV14 in the Rhizobiales

**Figure 3:**
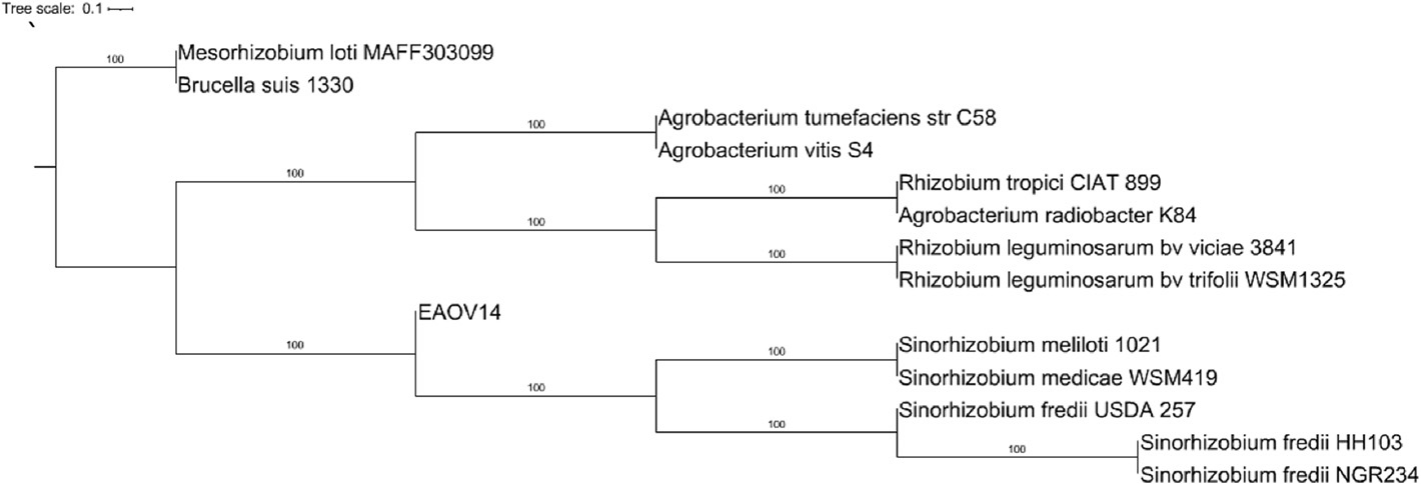
**Phylogenetic reconstruction based on the concatenated 16S rRNA, 23S rRNA, *atpD*, *chvA *(*ndvA*), *chvB*, *chvD*, *chvG *(*exoS*), *dnaK*, *exoC*, *gap*, *gyrB*, *infB*, *miaA*, *nusA pcs*, *pnp*, *recA*, *rpoB*, and *thrC *gene sequences.** Analyses were conducted using the consensus method (majority rule extended) with 100 bootstrap replicates. Bootstrap scores are represented numerically above branches. EAOV14 represents *E. adhaerens*.

By concatenating the full length sequence of 12 housekeeping (16S rRNA, 23S rRNA, *atpD*, *dnaK*, *exoC*, *gap*, *gyrB*, *infB*, *nusA*, *pnp*, *recA*, *rpoB*, *thrC*) and 8 rhizobial virulence genes (*chvA*(*ndvA*), *chvB*, *chvD*, *chvG*(*exoS*), *miaA*, and *pcs*) OV14 grouped with the *Sinorhizobium* of the *Ensifer*/*Sinorhizobium* group forming a clade which is a sister group to the *Rhizobium/Agrobacterium* clade. Within the *Ensifer*/*Sinorhizobium* group, *E. adhaerens* formed a sister group to the *Sinorhizobium* species (Figure 3). BLASTn searches of each replicon of OV14 revealed the highest synteny to *S. fredii* for the chromosomes and to *A. vitis* S4 pTiS4 and *A. tumefaciens* pTiC58 for the *E. adhaerens* pOV14 (Additional file 2). If considering the main chromosome, for which large scale synteny was observed, a gradient of sequence identity can be observed in OV14’s chromosome one; *S. fredii* strains shows 58% query coverage, *S. meliloti* strains shows 54% query coverage, *S. medicae* 49%, *Rhizobium* species including *A. rhizogenes* from 34–23%, and *A. tumefaciens* and *Mesorhizobium* species show 20% query coverage. Upon a BLASTn of the *A. tumefaciens* circular chromosome (Additional file 3) the query coverage to the closet matched rhizobia species was 34% dropping to 27%. Interestingly *S. fredii* species show higher coverage of the C58 circular chromosome at 26% than *A. vitis* does at 23%. Finally *S. meliloti* and *E. adhaerens* share 22% and 20% coverage with the *A. tumefaciens* C58 circular chromosome, respectively. Further support for this is available from the comparative NOG analysis, which reported OV14 as sharing more genes with 1021 than with C58, whilst also showing that OV14 shares more genes with C58 than 1021 and C58 share with each other (Figure 2).

## Discussion

While the ability of OV14 to genetically transform plant genomes has previously been demonstrated [12], OV14 has not acquired a known suite of symbiosis or pathogenesis genes but instead appears to effectively utilise supplementary *vir* or *sym* genes for virulence and symbiosis. For example; *E. adhaerens* strain ATCC 33499 was found to form nitrogen-fixing nodules on *Phaseolus vulgaris* (bean) and *Leucaena leucocephala* when equipped with the two symbiotic plasmids from *Rhizobium tropici* CFN299 [62], strain OV14 used in this study has shown the ability to deliver DNA into plants when equipped with the unitary pCambia5105 vector and strain 5D19 has been found to inhabit alfalfa nodules during a screen for diversity among the *S. meliloti* population [63]. Of interest, *R. etli* CE3 has been shown to possess *vir* gene homologs on a self-transmissible vector indicating a potential source of novel *vir* genes in soil bacteria [64].

For this study, the genome of OV14 was compared against the genomes of C58 and 1021 using eggNOG assignments to gain an understanding of their relatedness, with a focus being on an in/ability to transform plant cells. The potential of OV14 and 1021 to transfer T-DNA has only been achieved when equipped with a Ti-plasmid [7,12]. In this study we confirmed that there are no *Agrobacterium vir* gene homologs present in the OV14 genome. In the cases where homologs appeared in NOGs alongside *virB* and *virD4* it was likely due to parallel functions within type IV secretion systems. For the *virB* type IV secretion system *virB5*, *virB6*, *virB7* and *virB8* were not found as part of any NOG suggesting no similar genes in fully sequenced alphaproteobacteria to date. However, Sugawara et al. [45] detected five clades of type IV secretion systems within 48 sequenced *Sinorhizobium* species. Phylogenetic analysis found *A. tumefaciens virB* genes to be present in clade I with 1021 *virB* genes found in clade II along with *A. tumefaciens avh* genes. Interestingly, seven *Sinorhizobium* strains were found to possess type IV secretion systems in clade I [45].

Homologs to all chromosomal-based genes cited to be essential for T-DNA transfer in C58 were found to be present in OV14 and 1021, as indeed they are also present in several other alphaproteobacteria. Additionally all genes shown to be beneficial for *Agrobacterium* virulence were found in OV14, however not all of these genes were identified in 1021. At first it may appear that these genes are advantageous to life in the rhizosphere, however their presence in bacteria such as the animal pathogen *Brucella* broadens this hypothesis. Transgression into the rhizosphere or an animal host represents a dramatic change in environmental conditions. While nutrient availability may increase, changes in pH and eukaryotic cells defending themselves against invasion becomes a new challenge for the cell to overcome. Transcriptomic profiles in response to acidic pH (5.5) in both C58 and 1021 have shown the expression of genes involved in succionoglycan biogenesis and the regulation of acid inducible genes [42,65]. Down regulation of genes involved in motility via flagellum is also a shared response. Separately, electron microscopy analysis of OV14 completed by this research group has already confirmed the presence of functional flagella (Rathore et al. unpublished). The production of flagellum requires a large amount of energy and down regulation may free up energy for rearrangement of the cell envelope. Consequently, down regulation of flagellum may make the bacterial cell difficult to detect for the plant cell.

While many *exo* genes have been shown to be upregulated in C58 in response to acidic pH, the chvG/chvI sensor in C58 has not been shown to control their expression. Exerting control over cell envelope composition is most likely critical to the type of interaction that occurs with the plant cell directly via cell-to-cell contact or indirectly via the ability of the membrane to incorporate influential proteins and protein complexes. Indeed, differences in polysaccharide biosynthesis among *Sinorhizobium* species has been predicted as a host determination factor allowing for varying strategies for legume-*Sinorhizobium* interactions [45]. The C58 *aopB* has been shown to have a positive effect on virulence and was upregulated by acidic pH while the 1021 *aopB* homolog *ropB1* was not [42,66]. Of interest, a *ropB1 R. leguminosarum* bv. *viciae* VF39SM mutant was shown to have increased sensitivity to detergents, hydrophobic antibiotics, and weak organic acids, with a suggestion that *ropB1* plays a role in membrane stability [66]. For OV14, it would be of interest to complete a transcriptomic analysis to monitor the expression of these key genes under varying induction conditions.

Another cell wall component present in OV14, C58 and 1021 is phosphatidylcholine, a phospholipid and major component of eukaryotic membranes. Phosphatidylcholine has been shown to be essential for virulence in C58 and for symbiosis and normal growth in 1021 [67]. Interestingly an *A. tumefaciens* mutant deficient in phosphatidylcholine production was unable to support a type IV secretion system in the bacterial cell wall and subsequently lost its virulence potential [54]. The role of the virB type IV secretion system is known to be critical for T-DNA transfer to plants and is potentially the only system capable of this feat. Differences in phosphatidylcholine content in the cell wall of OV14, C58 and 1021 could affect the cells ability to support the complete virB type IV secretion system and greatly affect the transfer of T-DNA. The phosphatidylcholine synthase (pcs) pathway is choline-dependent and requires the uptake of choline into the cell. An *Agrobacterium pcs*/*pmtA* double mutant has been shown to be attenuated in expression of the *virB* operon [54] while a high-affinity choline ABC transport choXWV has been identified in C58 and 1021 [68,69]; OV14 was found to contain homologs to this system. The *choV* gene encodes the ATPase component of the ABC transporter. The fact that C58 has an additional copy of *choV* compared to OV14 and two additional copies of *choV* compared to 1021 suggests C58 may have an increased ability to actively acquire choline for phosphatidylcholine synthesis and ultimately complete T-DNA transfer into targeted host cells.

One of the most notable differences across the studied genomes was that OV14 and C58 possess *cel* homologs that are absent in 1021. The *cel* locus has been implicated in the attachment of *Agrobacterium* to the plant cell but is not required for tumour formation [70]. The presence of *cel* homologs in OV14 may suggest a role in attachment to plant surfaces or potentially other bacteria, which may explain its discovery within nodules alongside *S. meliloti* and the recorded ability of *E. adhaerens* to phagocytose other bacteria. Another observed difference was the lack of a type VI (T6SS) secretion system in OV14 and 1021 compared to C58. The lack of a type VI secretion system in 1021 was also noted by Sugawara et al. [45]. Type VI secretion systems are a relatively new discovery and their application is not well understood. However studies in *Vibrio cholerae* and *Pseudomonas aeruginosa* have shown the T6SS system to be involved in aggressive bacteria-to-bacteria cell interactions [71].

The chromosomal-based *acvB* gene has been cited as essential for *Agrobacterium* to achieve T-DNA transfer in the absence of *virJ* and has been found to localise to the periplasmic space and associate with the T-stand in *Agrobacterium*[72]. Homologs to the *acvB* gene were found in all three species in this study and another 25 alphaproteobacteria upon inspection of aproNOG05730. The homology of the two 1021 copies to the C58 *acvB* and OV14 homologs was found to be low, with it appearing that the 1021 *acvB* has split into two genes, which may be why *acvB* was not initially detected in 1021 [59]. The C58 and OV14 *acvB* shares 50% homology with *virJ* a gene found on octopine-type Ti plasmids which can complement a *acvB* mutant [73]. While acvB appears to play a role in export maybe as a chaperone to the T-strand [59,73], the OV14 *acvB* homolog shares 50% identity to its C58 counterpart and could be a key target for future studies focussed on the improvement of EMT.

The ability to defend against oxidative stress leads to increased virulence as the bacterial cell survives plant cell defences and acidic pH allowing expression of virulence genes and delivery of T-DNA to plant cell. All three species in this study were found to possess genes involved in protection against reactive oxygen species. One such gene that was absent in OV14 and 1021 was *dps*, (DNA-binding proteins from starved cells), which in *A. tumefaciens* protects the cell by acting as an hydroxyl radical scavenger and could well function with a catalase such as katA to increase the cell’s tolerance to the toxic effect of hydrogen peroxide [60].

In 1982, *E. adhaerens* was first described as a gram-negative predatory bacteria [13] but more recently, a request to rename *E. adhaerens* to *S. adhaerens* initiated a debate as to the appropriate nomenclature [74]. Following on from this the International Committee on Systematics of Prokaryotes ruled all *Sinorhizobium* species were to be transferred to *Ensifer* based on *Ensifer* being an early synonym of *Sinorhizobium*[13,75,76]. The current standing appears to be that *Ensifer* is the correct name for the amended genus, but the Judicial Commission also acknowledges the later synonym *Sinorhizobium*[76]. The work detailed here describes the genome sequencing of OV14 and based on a phylogenetic analysis of 20 housekeeping genes shows OV14 to form a branch separated from the *Sinorhizobia*. While the primary chromosome of OV14 shows a high level of synteny with the *Sinorhizobia* (highest to *S. fredii*) the remaining replicons share minimal synteny to any known species and are a potential resource of novel alpha-proteobacterial genes.

## Conclusions

This study has confirmed the presence of genes in OV14 that are confirmed homologs of chromosomal-based C58 virulence genes. As to how much their sequence diversity affects their function during T-DNA transfer remains unknown. Whereas the reported transformation efficiency of OV14 was achieved with environmental conditions optimal for *A. tumefaciens*, it is possible that Ti virulence induction conditions for non-*Agrobacterium* species may be different to *A. tumefaciens* and this is therefore an area that requires further attention. Re-engineering these non-*Agrobacterium* species with improved virulence functions offers the opportunity to increase the range of bacterial species that can be used for the genetic transformation of plant cells. Considering the limitations to the host range of *A. tumefaciens* have already been described [16,18,77], the use of non-pathogenic bacterial species may increase the range of plant species amenable to agronomic enhancement via genetic transformation.

## Methods

OV14 was originally isolated from the rhizosphere of *Brassica napus* at Oak Park in Carlow, Ireland. The strain was selected for sequencing based on its ability to transform plant cells [12].

### DNA isolation

Strain OV14 was grown to midlogarithmic phase in TY medium at 28°C, 200 rpm. DNA was isolated from 20 ml of cells using a modified CTAB (Cetyl thrimethylammonium bromide) genomic DNA isolation method [78]. RNase (20 mg/ml) was added to lysis buffer in step 3, and centrifugal spins were extended to 20 mins to allow separation to lysate and supernatant.

### Genome sequencing

The OV14 genome was sequenced and constructed by BaseClear B. V. Leiden, Netherlands. A hybrid approach using the Illumina HiSeq and PacBio RS platforms was selected. The genome was constructed from 1GB Illumina paired-end reads, 500 MB Illumina mate paired end reads, and 100 MB PacBio RS reads. The assembly was built in the following manner. First Illumina raw reads filtered using CASAVA version 1.8.2 and subsequently trimmed based on the Phred quality scores using the CLC Genomics workbench 1.8.3. Filtering of PacBio CLR reads was performed using the PacBio SMRT analysis suite. The quality-trimmed sequence reads were puzzled into a number of contig sequences with the CLCbio de novo assembler. This set defines the draft assembly. Subsequently the contigs were linked and placed into super-scaffolds based on the alignment against the long PacBio CLR reads. Alignment of the contigs was performed with BLASR [79]. From the alignment the orientation, order and distance between the contigs was estimated. As a result contigs were placed in super-scaffolds. This analysis was performed using a modified version of the SSPACE Premium scaffolder version 2.3 [80]. Finally gapped regions within the super-scaffolds were (partially) closed in an automated manner using GapFiller version 1.10 [81]. The method takes advantage of the insert size between the Illumina paired-end reads. The resulting scaffolds define the draft genome and plasmids, with the genome sequence available in the NCBI database under accession numbers CP007236.1, CP007237.1, CP007238.1 and CP007239.1.

### eggNOG analysis

Glimmer-predicted coding regions in the OV14 genome were BLASTp searched against an alphaproteobacteria database downloaded from eggnog.embl.de and assigned to NOGs based on similarity with a cut-off of 60 bits used to filter data. A reciprocal blast analysis (genome to EGGNOG and EGGNOG to genome) was also completed to ensure that recorded hits were evident in both directions, regardless of obtained low r values, which may have been due to evolutionary distinctness of the species. For comparative analysis all alphaproteobacterial gene families and their corresponding functional classifications were retrieved from eggNOG. The Functional categories used are based on: A Genomic Perspective on Protein Families [82]. The literature was screened for all genes known and predicted to be involved in T-DNA transfer and genes induced by the rhizosphere/rhizoplane environment across all NOG categories.

### Phylogenetic analysis

FASTA files for individual genes were obtained from NCBI and aligned using Clustal Omega. Clustal files were converted to Phylip format using an online tool found at http://insilico.ehu.es/tophylip/. Phylip files were concatenated using Seaview 4. The 40,470 base pair concatenated file was run using raxmlGUI producing a consensus tree with 100 bootstrap replicates. The tree was rooted by treating *Brucella suis* 1330 and *Mesorhizobium loti* MAFF303099 as the outgroup.

## Availability of supporting data

The following additional data are available with the online version of this paper. Additional file 1 includes Figures S1 and S2. Additional file 2 includes BLAST search scores for OV14 replicons. Additional file 3 includes BLAST search scores for C58 replicons.

## Abbreviations

EMT: *Ensifer*-mediated transformation; AMT: *Agrobacterium*-mediated transformation; Ti: Tumor inducing; T-DNA: Transfer-DNA; ROS: Reactive oxygen species; SA: Salicylic acid; T6SS: Type VI secretion system.

## Competing interests

The authors declare no competing interests.

## Authors’ contributions

SR, EM, FD, TW and CC developed the concepts and designed the research. SR performed the research and analysed the data with assistance from CC, EM and FD. EM and FD supervised the project. SR and EM prepared the paper. All authors edited read and approved the submitted manuscript.

## Supplementary Material

Additional file 1: Figure S1 Circular representation of the four replicons of *E. adhaerens* OV14. Circles, from the *inside out*, show: (1) GC skew; (2) Coding regions; light blue blocks represent genes with predicted function, red blocks show transposable elements, dark blue and grey blocks show genes of hypothetical and unknown function, respectively. **Figure S2.** Synteny plots showing total sequence of *Ensifer adhaerens* OV14 pOV14c (top bar) vs *Agrobacterium tumefaciens* C58 pTi (bottom bar), computed using DoubleACT version2 on tBLASTx setting with cut off set at 100. Visualised in Artemis ACT. Homology between the genomes is displayed via interconnecting lines; red lines representing direct homology while blue lines represent homologues but inverted sequence. Click here for file

Additional file 2 **BLAST of *Ensifer adhaerens* OV14 replicons.xlsx.** Excel file includes tables of BLAST search of individual *Ensifer adhaerens* OV14 replicons. Click here for file

Additional file 3 **BLAST of *Agrobacterium tumefaciens *** **C58 replicons.xlsx.** Excel file includes tables of BLAST searches of individual *Agrobacterium tumefaciens* C58. Click here for file
