# Supplementary material for: Genome sequence of Ensifer adhaerens OV14 provides insights into its ability as a novel vector for the genetic transformation of plant genomes
Source: BMC Genomics. 2014 Apr 7;15:268. doi: 10.1186/1471-2164-15-268 (PMC4051167; doi:10.1186/1471-2164-15-268)

**Additional file 1:**

**Figure S1**. Circular representation of the four replicons of *E. adhaerens* OV14. Circles, from the *inside out,* show: (1) GC skew; (2) Coding regions; light blue blocks represent genes with predicted function, red blocks show transposable elements, dark blue and grey blocks show genes of hypothetical and unknown function, respectively.

**Figure S2.** Synteny plots showing total sequence of *Ensifer adhaerens* OV14 pOV14 (top bar) vs *Agrobacterium* *tumefaciens* C58 pTi (bottom bar), computed using DoubleACT version2 on tBLASTx setting with cut off set at 100. Visualised in Artemis ACT. Homology between the genomes is displayed via interconnecting lines; red lines representing direct homology while blue lines represent homologues but inverted sequence.

**Additional file 2;** BLAST of *Ensifer adhaerens* OV14 replicons.xlsx. Excel file includes tables of BLAST search of individual *Ensifer adhaerens* OV14 replicons

**Additional file 3**. BLAST of *Agrobacterium tumefaciens* C58 replicons.xlsx. Excel file includes tables of BLAST searches of individual *Agrobacterium tumefaciens* C58

**Figure S1 Rudder et al**


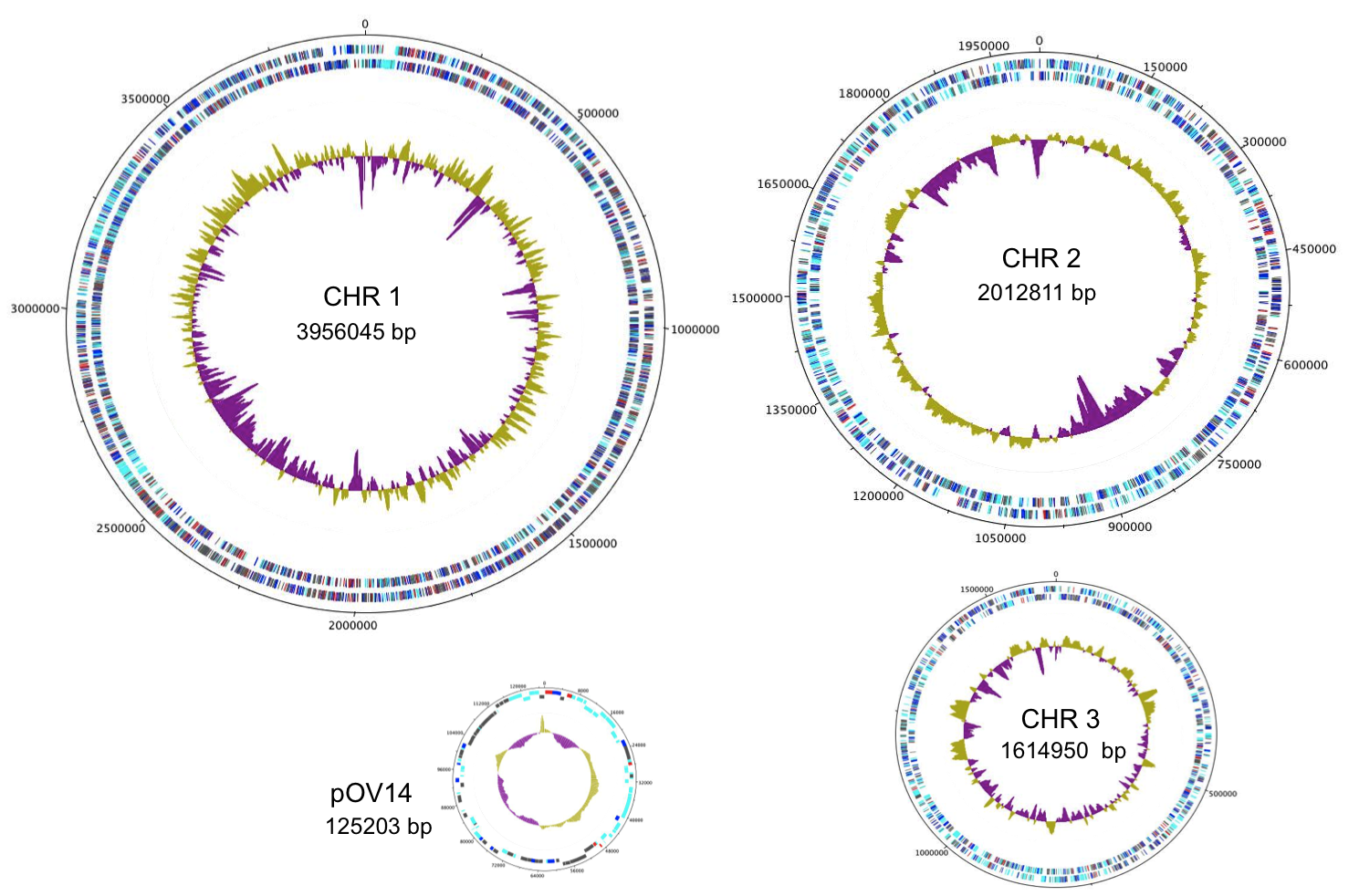


**Figure S2 Rudder et al**


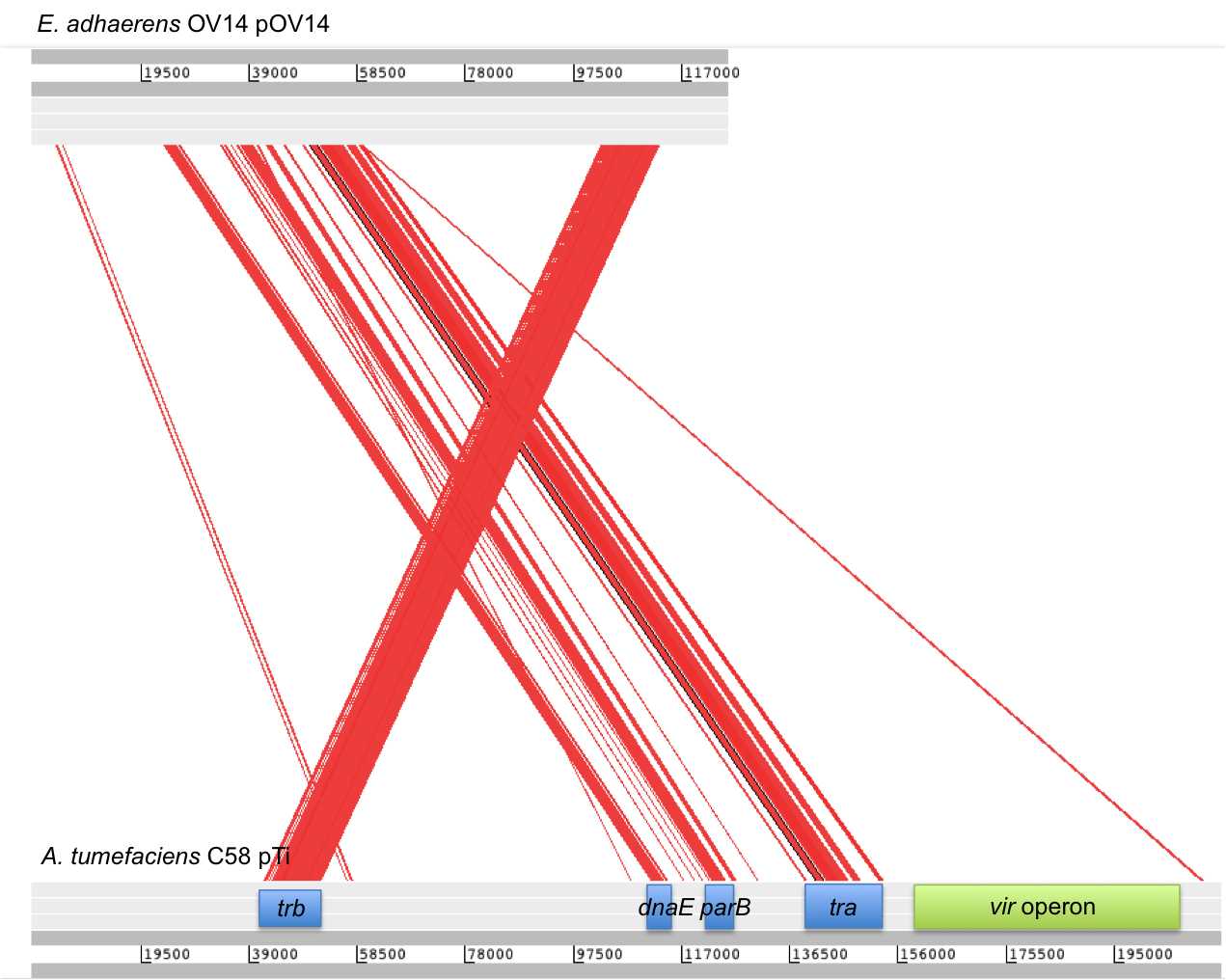

Supplement: Additional file 1: Figure S1 — Circular representation of the four replicons of E. adhaerens OV14. Circles, from the inside out, show: (1) GC skew; (2) Coding regions; light blue blocks represent genes with predicted function, red blocks show transposable elements, dark blue and grey blocks show genes of hypothetical and unknown function, respectively. Figure S2. Synteny plots showing total sequence of Ensifer adhaerens OV14 pOV14c (top bar) vs Agrobacterium tumefaciens C58 pTi (bottom bar), computed using DoubleACT version2 on tBLASTx setting with cut off set at 100. Visualised in Artemis ACT. Homology between the genomes is displayed via interconnecting lines; red lines representing direct homology while blue lines represent homologues but inverted sequence. [file 1471-2164-15-268-S1.DOCX]
